# Roasted and Unroasted Cocoa Nibs: Bioactive Compounds Analysis and Application in Cereal Bars

**DOI:** 10.3390/foods13213510

**Published:** 2024-11-02

**Authors:** Mariane Sampaio da Silveira de Souza, Larissa Lorrane Rodrigues Borges, Hélia de Barros Kobi, Valdeir Viana Freitas, Thaís Caroline Buttow Rigolon, Lary Souza Olegário, Eliana Alviarez Gutiérrez, Pedro Henrique Campelo Felix, Marcia Cristina Teixeira Ribeiro Vidigal, Paulo Cesar Stringheta

**Affiliations:** 1 Department of Food Technology, Universidade Federal de Viçosa, Avenue Peter Henry Rolfs, s/n, Viçosa 36570-900, Brazilpedrocampelo@ufv.br (P.H.C.F.); marcia.vidigal@ufv.br (M.C.T.R.V.); pstringheta@gmail.com (P.C.S.); 2 Department of Animal Production and Food Science, Universidad de Extremadura, 10003 Cáceres, Spain; 3 Research Institute for Sustainable Development of de Ceja de Selva, Universidade Nacional Toribio Rodríguez de Mendoza do Amazonas, Chachapoyas 01001, Peru

**Keywords:** cocoa nibs, phenolic compounds, methylxanthines, cereal bars, functional foods

## Abstract

Cocoa beans contain a variety of nutritional compounds and are rich in biologically active substances. The aim of this study was to utilize cocoa nibs (roasted and unroasted) as the main ingredient in the development of an attractive and convenient product. The produced nibs were analyzed for total phenolics, flavonoids, and antioxidant capacity using DPPH, ABTS, and FRAP methods. The primary phenolic compounds and methylxanthines were analyzed by LC/MS. Subsequently, cereal bars were developed, including a control sample (without nibs) and five formulations containing 41% nibs, using various proportions of roasted and unroasted nibs. The influence of the origin of the beans on the product characteristics was also evaluated. The results showed that the roasting process led to a reduction in epicatechin, caffeine, and caffeic acid. Furthermore, the reduction in total phenolics, flavonoids, and antioxidant capacity after roasting was more pronounced in beans from Bahia compared to those from Espírito Santo. Regarding the cereal bars, the results demonstrated that using cocoa from Bahia, the formulation with a higher proportion of unroasted nibs (F80) significantly increased the total phenolic content (1968.85 mg of gallic acid/100 g) and total flavonoids (39.26 mg of quercetin/100 g). This initial study suggests that the use of cocoa nibs as a functional ingredient in cereal bars may be a viable and advantageous option for creating a product with greater antioxidant potential.

## 1. Introduction

For a long time, cocoa consumption has been associated with improved health, and has become the focus of scientific research due to its health-promoting properties [1]. Cocoa is one of the richest natural sources of phenolic compounds and has high antioxidant potential [2]. 

In producing regions, cocoa cultivation mobilizes a large labor network and has a strong social, economic, and cultural impact. Currently, Brazil is the sixth largest producer in the world, and there is a growing demand for domestic consumption [3]. Production is primarily concentrated in the states of Pará, Bahia, and Espírito Santo [4]. Achieving consistent quality is a constant challenge, as cocoa beans are produced by a large number of independent farmers. In this context, Geographical Indication protects cultural heritage and artisanal production while providing identity to the products [5].

The processing of the beans leads to a significant change in the content of phenolic compounds and, consequently, in the biological activity of cocoa-derived products [6]. Each stage of cocoa processing alters the chemical composition of the bean, with sensory characteristics being primarily developed during the fermentation, drying, and roasting processes [7,8].

Roasting is a thermal process characterized by the reduction of moisture content, the decrease of undesirable volatile acids, and enzymatic inactivation, which primarily prevents the degradation of cocoa butter. It also involves the development of aromas through the Maillard reaction, utilizing the precursors formed during the fermentation stage [9]. Roasting induces chemical and physical changes that add value, with color, aroma, and texture being the most significant attributes [10].

Traditionally, cocoa beans are used to produce chocolate, cocoa butter, and cocoa powder. Recently, fermented, dried, and roasted beans have been marketed in small pieces known as cocoa nibs [11]. Nibs are a versatile product and can be consumed in smoothies, cakes, cookies, shakes, yogurts, among other items [12].

Cereal bars have been gaining prominence for combining innovation, convenience, and healthiness in a single food product. Due to their versatility, they are often considered multifunctional foods, used as travel snacks, meal replacements, and pre- or post-workout foods [13]. Cereal bars could be an alternative for incorporating cocoa nibs as a main ingredient, as they not only add a pleasant flavor to the product but may also enhance its nutritional quality.

There is a growing demand from consumers for healthy and convenient snacks. Data show that consumers tend to purchase snacks with functional claims. In 2022, 56% of Brazilians who consumed cereal bars stated that their consumption was due to the product’s nutritious and healthy characteristics [14]. 

Given the outlined context of the product’s market potential and the need for developing a healthier dietary option for consumers, this study aimed to develop cereal bars with cocoa nibs from different origins as the main ingredient. Additionally, the impact of roasting on the bioactive compound content and antioxidant activity of cocoa beans from Bahia and Espírito Santo was evaluated.

## 2. Materials and Methods

### 2.1. Chemicals and Reagents

All chemicals used in this study were of analytical grade. The following reagents and standards were purchased from Sigma Aldrich Co. (St. Louis, MO, EUA): Folin-Ciocalteu reagent, 1,1-Diphenyl-2-picrylhydrazyl (DPPH), 2,2’-Azino-bis (3-ethylbenzthiazoline-6-sulfonic acid) (ABTS), 6-Hydroxy-2,5,7,8-tetramethylchroman-2-carboxylic acid (Trolox), gallic acid, cyanidin-3-glucoside, sodium carbonate, acetonitrile, anhydrous sodium sulfate >99%, caffeine, (+)-catechin, (-)-epicatechin, caffeic acid, theobromine, p-coumaric acid, quercetin. Petroleum ether (30-70) PA-ACS 100%, hydrochloric acid (Synth, São Paulo, Brazil), potassium persulphate, potassium hydroxide 85% and hydrochloric acid 37% (Vetec Química Fina Ltd, Rio de Janeiro, Brazil), gallic acid (Analyticals Carlo Erba, France), methanol 99,8% (AlphaTec Systems, USA) and dichloromethane 99.5% (Química moderna Indústria e Comércio, São Paulo), absolute ethyl alcohol and acetone P.A. ACS (Êxodo científica, São Paulo, Brasil), acetic acid P.A. (ISOFAR ltda., Rio de Janeiro, Brasil), PVDF Whatman qualitative filter paper, Grade 1 (Merck, Darmstadt, Germany). Aqueous solutions were made with Milli-Q water (18.2 mΩ) (Millipore, Bedford, MA, USA).

### 2.2. Samples

The beans of the Forastero variety were acquired in 2021 during the months of May and June, from age plants approximately 40 years old, and were collected in two regions relevant to cocoa production in Brazil. One of the samples was collected in Bahia and has a designated origin (14°41′21.1″ S and 39°17′02.7″ W). The other sample was produced in Espírito Santo, in the city of Linhares (19°21’23.1” S and 40°13’33.5” W). The fruits underwent primary processing stages (fermentation and drying) on the farms where they were harvested.

The cocoa beans were vacuum-packed and stored for 15 days in a cool (approximately 25 °C) and dry location until the preparation of the cereal bar and subsequent analyses.

### 2.3. Sample Characterization and Processing

The cocoa beans were selected and separated from the shell, and subsequently evaluated for their physicochemical characteristics of pH, moisture, and fermentation index (FI). 

#### pH, Moisture and Fermentation Index

The AOAC (Association of Official Analytical Chemists) reference method was employed for pH determination. The pH was measured in an aqueous sample suspension using a digital pH meter (Digimed^®^, model DM-20, Digicrom Analytical Ltd., São Paulo, Brazil) [15]. For moisture determination, the direct drying method in an oven at 105 °C was used, following the analytical standards proposed by the Manual do Instituto Adolfo Lutz [16]. The fermentation index was calculated following the method described by Gourieva & Tserevitinov [17]. Ground cocoa beans (0.5 g) were added to 50 mL of a solution of methanol and hydrochloric acid (97:3, *v*/*v*). The mixture was then stored under refrigeration (8 °C) for 18 h. This mixture was filtered through Whatman No. 1 filter paper, and the filtrate was read on a UV-M51 spectrophotometer (Bel, Monza, Italy) at wavelengths of 460 nm and 530 nm. FI was calculated as the ratio of the absorbance of oxidized and polymerized anthocyanidins (460 nm) to that of anthocyanin monomers (530 nm), as described in Equation (1).

(1) FI=A460A530

### 2.4. Nibs Unroasted and Roasted

To obtain the unroasted beans, the shell was manually removed, and they were crushed in a mixer (Mallory, Trikxer Pratic model, São Paulo, Brazil) for about three minutes. 

For the roasted beans, they were placed whole in a oven at 135 °C for 35 min (SolidSteel SSD, São Paulo, Brazil) following the method proposed by [5], in order to preserve the highest possible content of phenolic compounds. After roasting, the beans’ shells were removed, and for the preparation of the nibs, a mixer (Mallory, Trikxer Pratic model, São Paulo, Brazil) was used for about 3 min. The particle size of the nibs (roasted and unroasted) used in the formulation of the cereal bars was less than 3.36 mm.

### 2.5. Preparation of Cereal Bars

The cereal bars were developed following the formulations suggested by Lima et al. [18] and Carvalho and Conti Silva [19], with some modifications. The amount of cocoa added to the cereal bars was based on Farah’s [20] classification for chocolates, using cocoa content equivalent to that of semisweet dark chocolate (40–55%). The proportion of dry ingredients and syrup (binding agents) was determined through preliminary tests. The ingredients used are listed in Table 1, all were purchased in local markets in the city of Viçosa, Minas Gerais, Brazil.

A control formulation was prepared without the addition of cocoa. In the other formulations, 41% of cocoa nibs were added with varying proportions of roasted and untoasted nibs. All ingredients were weighed using an analytical balance, and subsequently, the syrup ingredients (honey, cane molasses, brown sugar, soy lecithin, salt, and water) were heated on a hot plate at 160 °C and stirred for about 8 min to melt the sugars.

**Figure 1:**
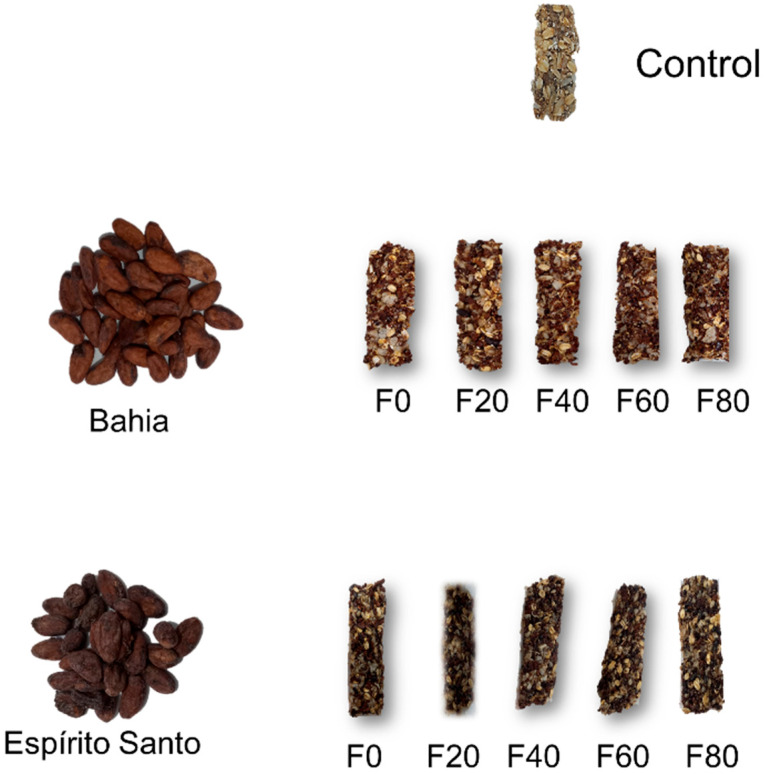
Formulated cereal bars. The composition of the formulations (F0, F20, F40, F60, and F80) are described in Table 1.

The dry ingredients were mixed with the syrup and homogenized using a stainless-steel spoon. The resulting dough was placed into a glass mold lined with parchment paper and pressed to form the product shape. After cooling, the cereal bars were cut into uniform sizes and individually packed in flexible film packaging at room temperature (approximately 25 °C). Figure 1 shows the cocoa beans of different origins and the formulated cereal bars. 

The cereal bars were packaged in flexible film and maintained at a temperature of approximately 25 °C. The following day, they were evaluated for water activity, texture, color, total phenolic, and flavonoid content, as well as antioxidant capacity.

### 2.6. Analysis of Bioactive Compounds

For the analysis of total phenolic compounds, total flavonoids, and antioxidant capacity, it was necessary to obtain extracts. These analyses were performed for both roasted and unroasted cocoa nibs, as well as for all formulated cereal bars.

Firstly, the samples were grounded in a porcelain mortar and defatted according to the method described by Quiroz Reyes and Fogliano [21], with some modifications. To the powder obtained after maceration, petroleum ether (at a ratio of 1:4 *w*/*v*) was added and heated at 40 °C in a water bath for 15 min. After this period, the mixture was stirred using a horizontal shaker (Kliner, Nova Técnica, São Paulo, Brazil) for 35 min. The mixture was then filtered through Whatman No. 1 paper, and the resulting defatted powders were placed in an oven at 40 °C until the complete evaporation of the petroleum ether.

The extracts were obtained following the procedure proposed by Counet and Collin [22], with some modifications. Ten grams of the defatted powder were used in 50 mL of an extraction solution containing acetone, water, and acetic acid (70:28:2 *v*/*v*). This mixture was subjected to ultrasonic treatment at 25 kHz and 25 °C for 1 h using an ultrasonic bath (Elmasonic TI-H-10, Elma, Germany). Subsequently, the mixture was filtered through Whatman No. 1 paper, and the resulting extracts were stored in a freezer (approximately −18 °C) until analysis. 

#### 2.6.1. Total Phenolic Compound Content

Following the methodology proposed by Singleton and Rossi [23], the determination of total phenolic compounds was carried out using the Folin–Ciocalteu reagent. The absorbance of the samples was measured at a wavelength of 760 nm using a UV-M51 spectrophotometer (Bel, Monza, Italy). Gallic acid was used as a standard, and the results were expressed as mg of gallic acid equivalent per 100 g.

#### 2.6.2. Total Flavonoid Content

The concentration of total flavonoids was determined following the method described by Arvouet-Grand et al. [24], using a reaction with aluminum chloride (AlCl_3_). Quercetin was used as the standard, and the spectrophotometric readings (UV-M51, Bel, Monza, Italy) were performed at 425 nm. The results were expressed as mg of quercetin equivalent per 100 g.

### 2.7. Determination of Antioxidant Capacity

#### 2.7.1. ABTS Method

The ABTS method was performed as described by Re et al. [25], with some modifications. To form the ABTS˙⁺ radical, an aqueous solution of 7 mM ABTS was reacted with a 2.45 mM potassium persulfate solution in a 1:1 ratio, in the absence of light, and allowed to stand for 16 h at room temperature. At the time of analysis, this solution was diluted with 80% ethanol until an absorbance of 0.700 was reached at 734 nm using a spectrophotometer (UV-M51, Bel, Monza, Italy).

The ABTS˙⁺ solution was added to the sample, diluted in 5 increasing concentrations, or to standard Trolox solutions for constructing the analytical curve. The reaction occurred in the absence of light for 6 min. The results were calculated using the analytical curves of the samples and expressed as Trolox equivalent antioxidant capacity (µM TEAC/g).

#### 2.7.2. DPPH Method

The DPPH method was performed according to Kim et al. [26] with modifications. In summary, 0.5 mL of each sample extract was mixed with 3.5 mL of the DPPH solution, and the reading was taken using a spectrophotometer (UV-M51, Bel, Monza, Italy) after 1 h of reaction. Trolox was used as the standard for the analysis, and the results were expressed as Trolox equivalent antioxidant capacity (µM TEAC/g).

#### 2.7.3. FRAP Method

The antioxidant capacity was assessed using the FRAP assay method according to Laurrauri, Rupérez, and Saura-Calixto [27]. For the analysis, 90 µL of the sample, 270 µL of distilled water, and 2.7 mL of the FRAP reagent (a mixture of 0.3 mol/L sodium acetate buffer, 10 mmol/L TPTZ (2,4,6-tris(2-pyridyl)-s-triazine), and 20 mmol/L ferric chloride solution in a 10:1:1 ratio) were added. This mixture was incubated in the absence of light for 30 min at 37 °C, and the absorbance was then measured at 595 nm using a spectrophotometer (UV-M51, Bel, Monza, Italy). An analytical curve was prepared with ferrous sulfate, and the results were expressed as ferrous sulfate equivalent antioxidant capacity (µM FSE/g). 

### 2.8. Analysis of Phenolic Compounds and Methylxanthines by Targeted LC/MS

Approximately 400 μL of the extracts were placed in vials, and 5.0 μL were injected into the LC/MS system from NuBioMol (Center for Biomolecules Analysis-UFV, Brazil) using a chromatography column (Agilent Eclipse Plus, RRHD, 1.8 μm, 2.1 × 150 mm) with a flow of 0.25 mL min^−1^, coupled online to a mass spectrometer QQQ triple quadrupole (Agilent). The mobile phase consists of buffers A (water 100% and acetic acid 0.02% pH 4.5) and B (acetonitrile 100% and acetic acid 0.02% pH 4.5), and the following gradient program was used: a linear ramp starting at 2% of A, increasing to 19% of B for 5 min, 60% of B for 13 min, and 98% of B at 14 min; 98% of B was maintained during 3 min (17 min), and a descent linear gradient starting at 98% of B, decreasing to 2% of B by 1 min (18 min), followed by an equilibrium condition at 2% of B for 2 min. 

The mass spectrometer was operated in negative/positive alternating modes, and the sample was scanned by multiple reaction monitoring (MRM) using the mass transitions for phenolic compounds. The mass spectra generated were processed using Skyline software (64 bit version 24.1) to obtain the extracted ion chromatograms (EIC) of each transition and the chromatogram area values from EIC. Standard curves for the compounds (epicatechin, catechin, quercetin, caffeine, theobromine, caffeic acid, and p-Coumaric acid) were used to convert peak area values into ng/g of cocoa.

### 2.9. Colorimetric Analysis

The color of the cereal bars was determined using a Colorimeter ColorQuest XE (Hunter Lab, Reston, VA, USA) calibrated according to the manufacturer’s instructions, under the D65 illuminant and at a 10° observation angle, with specular reflectance included (RSIN). Readings were performed in the CIELAB system, measuring the coordinates L* (lightness), a* (red vs. green intensity), and b* (yellow vs. blue intensity). The hue (h*) and chroma (C*) parameters were calculated from the a* and b* values, according to Equations (2) and (3), respectively. The overall color difference was also calculated between the sample containing only roasted cacao nibs and the other bars containing unroasted cacao nibs in different concentrations, according to Equation (4), in order to understand if the addition of unroasted nibs would cause a significant change in coloration.

(2) $$h^{*}=arctan\left(\frac{b^{*}}{a^{*}}\right)$$

(3) $$C^{*}=\sqrt{\left({a^{*}}^{2}+{b^{*}}^{2}\right)}$$

(4) $$∆E^{*}=\sqrt{{\left(∆L^{*}\right)}^{2}+{\left(∆a^{*}\right)}^{2}+{\left(∆b^{*}\right)}^{2}}$$

### 2.10. Instrumental Texture

Instrumental texture analysis was conducted using a Texture Analyzer (Brookfield CT3, Middelboro, MA, USA) with a knife blade to measure shear force for hardness determination. The dimensions of the analyzed cereal bars were 20 × 20 mm and 10 mm in thickness. The device was configured with the following parameters: pre-test, test, and post-test speeds of 2.0 mm/s and a distance of 10.0 mm. The test was performed in five repetitions. 

### 2.11. Water Activity

The water activity of the formulated cereal bars was measured by direct reading using a water activity meter (AquaLab 4TE, Decagon Devices, Pullman, WA, USA) at a temperature of 25 °C.

### 2.12. Statistical Analyses

All analyses were conducted in triplicate, and the data were expressed as means ± standard deviations (SD). Moisture, pH, and fermentation index data were analyzed using the *t*-test. The chemical composition of the samples was evaluated by two-way ANOVA, followed by Tukey’s test for multiple comparisons, considering a 2 × 2 factorial design. The first factor was cocoa origin (Bahia and Espírito Santo), and the second was roasting (roasted or unroasted). Data on bioactive compounds and antioxidant activity of the cereal bars were analyzed by two-way ANOVA, followed by Tukey’s test for multiple comparisons, considering a 2 × 2 factorial design. The first factor was the different formulations (Control, F0, F20, F40, F60, F80), and the second was roasting (roasted or unroasted). Physicochemical parameters (hardness and water activity) were analyzed by one-way ANOVA, followed by Tukey’s test. All analyses were performed using R software version 4.3.3 (R Core Team, Vienna, Austria). A *p*-value < 0.05 was considered statistically significant.

## 3. Results and Discussion

### 3.1. Characterization of Cocoa Beans from Different Origins

During fermentation, microorganisms produce acids that lower pH values. These parameters are often used to assess the quality of dried cocoa beans. Unroasted beans from Bahia and Espírito Santo were analyzed for fermentation index, pH, and moisture content, with the results presented in Table 2.

Fermentation is primarily carried out using traditional methods, involving spontaneous fermentation based on local practices. An index value ≥ 1 indicates that the cocoa has been sufficiently fermented. Both samples evaluated in this study have values above 1. However, other factors such as pH must be considered to determine if the fermentation was effective.

The cocoa beans from Espírito Santo exhibited a lower pH compared to the Bahia cocoa samples. It is important to note that the Bahia beans used in this study are certified with the geographical identity of Southern Bahia, a certification regulated in 2016 to better standardize the beans from this [5]. Mougang et al. [28] suggested that for commercially viable cocoa beans of good quality, the pH should be between 5 and 5.5. 

Batista et al. [29] analyzed 78 cocoa samples from Bahia, with pH values ranging from 4.4 to 6.7, and the values obtained in this study fall within this range. Quiroz-Reyes and Fogliano (2018) [21] found pH values of 5.6 ± 0.01 for Forastero cocoa, while Gutierrez et al. [30] found pH values above 5.5 in fine-aroma native cocoa from Peru. According to the study conducted by Yusep et al. [31], cocoa beans with a pH close to 5.8 after fermentation are likely to produce special flavors and aromas in the cocoa. 

Primary processing ends with the drying of the beans, which is usually carried out by the producers themselves. The moisture content of the beans was 6.11% and 6.63% for the samples from Bahia and Espírito Santo, respectively. According to Efraim et al. [9], it is recommended that cocoa beans reach a moisture content of less than 8.0% and a water activity of less than 0.7, as this ensures physical, chemical, and microbiological stability during storage.

Roasting contributes to important sensory characteristics in cocoa. In this study, the roasting was done at 135 °C for 35 min, as proposed by Żyżelewicz et al. [8]. After roasting, the moisture content of the beans decreased to 2.97 ± 0.052% for the samples from Bahia and to 3.23 ± 0.070% for the beans from Espírito Santo.

### 3.2. Analysis of Roasted and Unroasted Cocoa Beans

Table 3 presents the results obtained for the total phenolic and flavonoid content in both unroasted and roasted cocoa beans.

As expected, the roasting of cocoa resulted in a significant loss of phenolic compounds due to their sensitivity to high temperatures. The reduction in total phenolics and flavonoids found in this study was 68.51% and 53.06% for the Bahia cocoa, and 23.78% and 7.31% for the cocoa from Espírito Santo. Only the total flavonoid content of the Espírito Santo cocoa did not show a significant difference after the roasting process.

According to Efraim et al. [32], cocoa beans contain approximately 6 to 8% phenolic compounds on a dry weight basis, with (+) catechin, (−) epicatechin, and procyanidins representing about 60% of this content. During roasting, the loss of these phenolic compounds is attributed to oxidation into corresponding quinones, which allows for polymerization and the formation of high molecular weight insoluble pigment compounds. Additionally, their reaction with proteins may contribute to the reduction of phenolics [33]. The high phenolic content found in the unroasted samples can be attributed to the use of the Forastero cocoa variety. This variety is characterized by dark brown beans with a mild aroma, robust flavor, moderate acidity, and high levels of cocoa butter and phenolic compounds [34].

Studies conducted by Oracz and Nebesny [35] on five different cocoa varieties, evaluating roasting at various temperatures (150 °C for 25 min; 135 °C for 40 min; 120 °C for 75 min; and 110 °C for 85 min) and relative air humidity during drying with variations of 0.2%, 2%, and 5%, observed that Brazilian Forastero cocoa exhibited the highest total phenolic content, with all samples being influenced by the roasting conditions.

Ioannone et al. [36], studying the effect of roasting on the content of flavonoids and proanthocyanidins and on the antioxidant activity of cocoa beans, observed that the rates of loss of flavanols and total proanthocyanidins increased with higher roasting temperatures (125, 135, and 145 °C) when the roasting time was kept constant. By setting the final moisture content to 2 g/100 g, high-temperature and short-time processes minimized the loss of proanthocyanidins. 

Polyphenols can exert their antioxidant power in the body through various mechanisms. Although in vitro techniques do not show biological effects, they provide a good approach to understanding the antioxidant effect of these extracts and their chemical mechanism of action. The antioxidant capacity values measured by the methods ABTS, DPPH, and FRAP for the cocoa beans studied are presented in Table 4.

Overall, there was a reduction in antioxidant capacity after roasting across the tested methods. However, in the DPPH assay, there was no significant difference between the unroasted and roasted beans from Espírito Santo.

Studies conducted by Oracz and Nebesny [35] also exhibited a similar pattern of scavenging activity to that observed in this work, with higher values obtained in the ABTS assay compared to the DPPH assay. For unroasted Forastero cocoa beans from Brazil, the authors found 1406.77 µM TEAC/g and 1370.12 µM TEAC/g for ABTS and DPPH, respectively.

Similarly to the content of phenolic compounds and total flavonoids, the reduction in antioxidant capacity, for all the studied methods (ABTS, DPPH, and FRAP), after roasting was more pronounced in the samples from Bahia.

### 3.3. Identification of Compounds by LC/MS

The main phenolic compounds and methylxanthines present were analyzed in the samples by LC/MS. The results are expressed in Table 5.

The main type of polyphenols in cocoa are flavanols, with epicatechin being the most abundant in the fruit, constituting about 35% of the total polyphenol fraction. Among the monomeric flavanols, epicatechin has been identified as the active compound responsible for the vascular health benefits associated with cocoa and chocolate [37,38]. Epicatechin was the most abundant compound in the present study; however, there was no significant difference regarding the origin of the beans.

During heating treatment, epicatechin undergoes epimerization reactions, which results in a decrease of this compound [39]. After the roasting process, its value decreased by approximately 16%.

Catechin and quercetin did not differ based on the origin of the beans. For catechin, there was no significant difference after the roasting treatment. Studies by Żyżelewicz et al. [8] indicate that during the first 15 min of roasting at 135 °C, there is a rapid increase in this compound, but its content decreases throughout the process, which can be explained by changes in the distribution of procyanidins. In this study, after the roasting process, an increase in quercetin concentration was also observed. 

Phenolic acids are also a source of antioxidants in the final products derived from cocoa. In the present study, caffeic acid differed with respect to the treatment, with roasting reducing its concentration by approximately 32%. Additionally, p-Coumaric acid varied based on the origin of the beans, with higher concentrations found in those from Espírito Santo.

According to Lemarc et al. [40], it is difficult to identify a clear trend regarding the content of phenolic acids in cocoa bean processing. Although these molecules are easily volatilized due to thermal degradation from roasting temperatures, they may also exhibit increased levels due to the dissociation of these compounds into larger molecules, such as proteins associated with phenolic acids. 

In addition to polyphenols, cocoa beans are also rich in methylxanthines, such as theobromine and caffeine, which have stimulating effects on the myocardium. The concentrations of theobromine and caffeine in cocoa beans are considered important factors determining cocoa quality, as they are related to sensory characteristics [41]. A low theobromine-to-caffeine ratio is commonly found in fine cocoa beans [42]. In this study, there was no significant difference in the area of these compounds based on the origin of the cocoa beans. The effect of roasting was significant only for caffeine, reducing it by approximately 18%. Generally, the caffeine content in cocoa beans decreases with longer roasting times due to sublimation and decomposition at high temperatures [43].

### 3.4. Cereal Bars with Added Roasted and Unroasted Nibs

**Figure 2:**
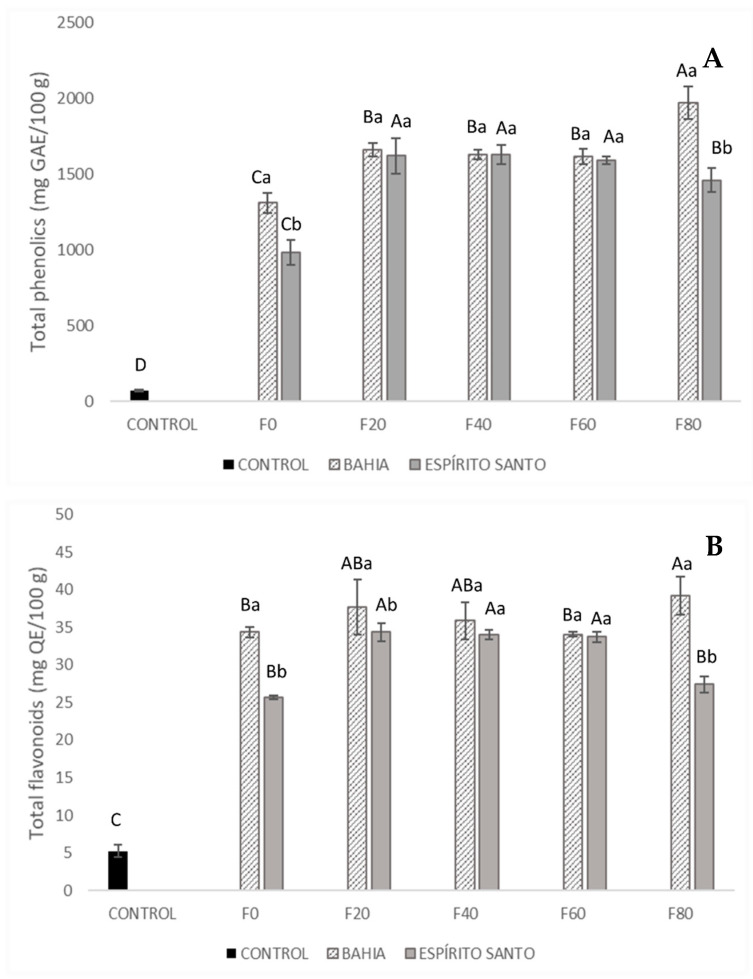
Total phenolic (**A**) and total flavonoid (**B**) content in cereal bars. Means followed by uppercase letters indicate that there are no significant differences regarding the formulation. Lowercase letters indicate that there are no significant differences concerning the origins according to Tukey’s test (*p* > 0.05). The composition of the formulations (F0, F20, F40, F60, and F80) is described in Table 1.

Each type of cereal bar has different characteristics and purposes that align with the trend of consuming healthy, innovative, and convenient foods, which has led to gradual growth in the cereal bar market. In the present study, cereal bars were developed as an alternative to the use of cocoa nibs, as well as to create a product with a high content of bioactive compounds. Regarding the content of phenolics and total flavonoids, the values are shown in Figure 2.

According to the results shown in Figure 2, all cereal bars formulated with the addition of nibs had significantly higher contents of phenolics and total flavonoids compared to the control cereal bar. Regarding the use of roasted and unroasted nibs, the F0 formulation (containing only roasted nibs) had lower values of phenolics and total flavonoids compared to the other formulations. In the cereal bars made with cocoa from Bahia, there was a significant difference in the total phenolic content of the sample containing only roasted nibs (F0), which was 1310.84 mg of gallic acid/100 g. The sample with the highest concentration of unroasted nibs (F80) also differed from the other cereal bars, with 1968.85 mg of gallic acid/100 g.

As previously observed, there was greater degradation of these phenolic compounds following the roasting process of the beans, with a more pronounced reduction in the samples from Bahia. In the cereal bars made with cocoa from Espírito Santo, no significant difference was observed regarding the content of unroasted nibs used. Only the formulation containing only roasted nibs (F0), which had 982.47 mg of gallic acid/100 g, differed significantly from the others.

It is important to note that the Folin–Ciocalteu assay is based on an oxidation–reduction reaction. Thus, the phenolic content measured by this method can be influenced by the presence of other non-phenolic reducing compounds, such as carbohydrates, pigments, and/or Maillard reaction products [44].

Regarding the total flavonoid content, no increase was observed when higher concentrations of raw nibs were added, both for cacao from Bahia and for cacao from Espírito Santo. The total flavonoid levels ranged from 34.04 to 39.26 mg of quercetin/100 g in cereal bars with cacao nibs from Bahia and from 25.69 to 34.35 mg of quercetin/100 g in cereal bars with cacao nibs from Espírito Santo. 

**Figure 3:**
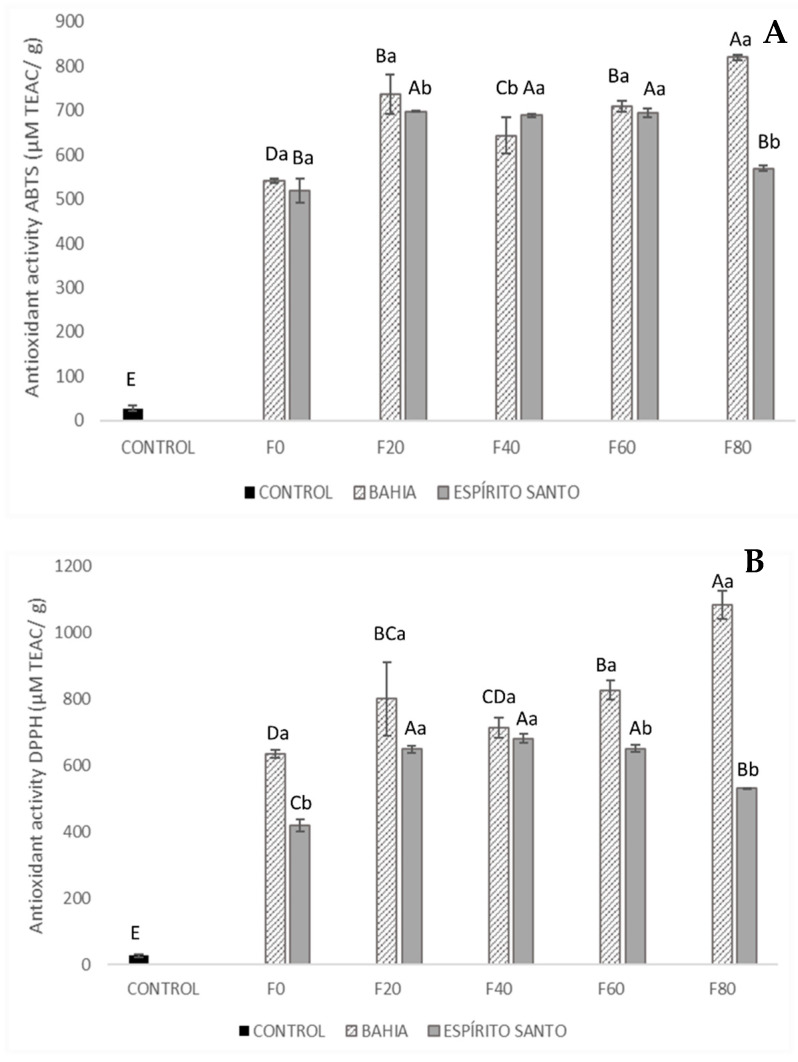

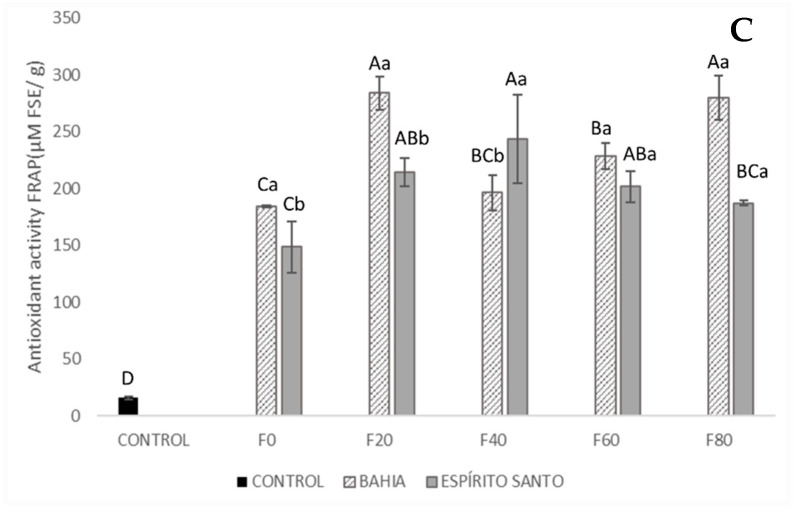
Antioxidant activity in cereal bars: (**A**) method ABTS, (**B**) method DPPH, and (**C**) method FRAP. Means followed by uppercase letters indicate that there are no significant differences regarding the formulation. Lowercase letters indicate that there are no significant differences concerning the origins according to Tukey’s test (*p* > 0.05). The composition of the formulations (F0, F20, F40, F60, and F80) is described in Table 1.

The results of the antioxidant capacity measured in the cereal bars are shown in Figure 3.

Consistent with the results regarding the total phenolic and flavonoid content, the control cereal bars exhibited significantly lower antioxidant activity compared to the cereal bars with added roasted and unroasted cocoa nibs.

In the cereal bars containing nibs from Bahia, a higher antioxidant capacity was observed in those with a greater concentration of unroasted cocoa nibs F80, showing 819.30 µM TEAC/g in the ABTS method and 184.43 µM TEAC/g in the DPPH assay. In the ABTS and FRAP assays, the formulation containing only roasted nibs F0 exhibited lower antioxidant capacity, with 541.59 µM TEAC/g and 183.91 µM FSE/g, respectively, compared to the other formulations.

For the formulations containing cocoa nibs from Espírito Santo, the same trend could not be observed. The values for the ABTS assays ranged from 518.09 to 697.63 µM TEAC/g, for the DPPH assays from 420.07 to 681.80 µM TEAC/g, and for the FRAP assays from 148.47 to 243.15 µM FSE/g.

The antioxidant capacity of the extracts from the cereal bars with added cocoa nibs can be attributed to the presence of components other than phenolic compounds, such as methylxanthines and/or Maillard reaction products, as well as interactions between these compounds [35].

Żyżelewicz et al. [34], in their study on chocolate made with different blends of cocoa liquor prepared from roasted and unroasted beans of the Forastero and Criollo varieties, observed that the type of liquor induced slight changes in the polyphenol composition. The liquor from roasted beans was characterized by a higher ability to scavenge radicals using the DPPH method.

The incorporation of other nutritious ingredients beyond cocoa can effectively enhance the functional properties of cereal bars. Therefore, it is important to highlight that the formulation of these bars included other ingredients with functional properties. For example, honey, a natural and sweet food product, is known for many medicinal and biological properties, including antioxidant, antibacterial, and anti-inflammatory functions [45,46]. 

The use of oats in processed foods is advantageous because they contain fibers, unsaturated fatty acids, various vitamins, and minerals, as well as having a well-balanced protein composition [47]. In the bars formulated in this study, 10% rolled oats and 6% fine whole oats were used.

It was observed that the cereal bars enriched with cocoa nibs exhibited significantly higher concentrations of phenolic compounds, flavonoids, and antioxidant activity compared to the control bar. The total phenolic content in the bars containing cocoa nibs was approximately 28 times higher than that in the control cereal bar (without cocoa nibs). 

Considering that the objective of the study was to develop a product with high phenolic content, the overall color difference was conducted exclusively on the samples containing cocoa nibs to assess the impact of adding unroasted nibs on the product’s coloration. The results obtained from the colorimetric analyses of the cereal bars with added nibs are listed in Table 6.

The colorimetric values of the cereal bar samples were all positive, indicating that they are located in the first quadrant of the colorimetric plane. The luminosity values range from 0 (black) to 100 (white), with the cereal bars with cocoa nibs exhibiting low luminosity values, indicating that the samples are darker, unlike the control sample, which showed a value of 59.80. The lowest values were found in the bars containing only roasted nibs, with values of 30.23 and 35.50 for the bars made with nibs from Bahia and Espírito Santo, respectively.

The saturation index (C*) corresponds to the length of the vector in the projection of the color location on the (a*, b*) plane, while the coordinate h* represents the hue angle [48]. In the cereal bars produced, both C* and h* values were low, indicating that the color is less saturated and has darker hues. 

Most of the pigments in cocoa beans are formed during fermentation and drying, so the initial color of the nibs before roasting is already dark brown, which complicates the visual color analysis for differentiating between roasted and unroasted nibs [49]. Therefore, the overall color difference (∆E*) was calculated by comparing the cereal bar samples containing roasted almond nibs (F0) with other samples containing different concentrations of unroasted almond nibs. Values below 5.0 were found only in the cereal bars containing lower concentrations of unroasted almond nibs F20 and F40, both for cocoa from Bahia and for cocoa from Espírito Santo. According to Ramos and Gomide [50], a global color difference above 5.0 is perceptible to the human eye.

These results from the global color difference in the cereal bars indicate that, regardless of the cocoa origin, the formulations produced exhibited similar behavior. Considering this result, along with the fact that cocoa nibs from Bahia showed higher values of analyzed bioactive compounds, which is desirable for cereal bars, we selected the cereal bars with cocoa nibs from Bahia to evaluate water activity and instrumental texture. These are important parameters for the final product, and the results are shown in Table 7.

Regarding hardness, the results ranged from 3.14 to 12.66 N. Hardness refers to the maximum shear force of the product and is related to the force required for penetration by the incisors, perceived at the moment of cutting. The hardness value of the control sample did not differ significantly from the other formulations evaluated, indicating that the addition of nibs (roasted and unroasted) did not alter this parameter.

Regardless of the origin of the cocoa beans or the concentration of unroasted cocoa nibs, the bars exhibited water activity below 0.6000, indicating that they are microbiologically stable.

## 4. Conclusions

Due to the increasing concern for healthy eating, the industry is striving to provide foods with higher nutritional value. Incorporating functional ingredients into existing food formulations is a viable alternative. This study demonstrated that cereal bars with the addition of cocoa nibs as a major ingredient can be a viable option for marketing a product with high levels of phenolic compounds, total flavonoids, and antioxidant capacity.

The roasting of cocoa beans resulted in a significant reduction of bioactive compounds, particularly in samples from Bahia. The innovation of incorporating unroasted nibs into cereal bars not only simplifies the processing by reducing one step but also prevents the loss of bioactive compounds. This study demonstrated that this addition did not significantly compromise the attributes of water activity and instrumental texture. All formulations with cocoa nibs demonstrated advantages compared to the control, with the total phenolic content, for example, reaching values 14 to 28 times higher than the control formulation.

It is noteworthy that, due to the ease of processing this product, these cereal bars can be produced by the cocoa farmers themselves, creating more business opportunities and strengthening social organizations in the fruit-producing regions. Further studies will be conducted to gather more information on the composition and sensory attributes of this product. However, considering that the global market for cereal bars has shown growth trends, it is likely that these products will be well received.

## Figures and Tables

**Table 1:** Formulations of the cereal bars.

Ingredients (g)		Formulations				
	Control	F0	F20	F40	F60	F80
Unroasted cocoa bean nibs	-	-	8.2	16.4	24.6	32.8
Roasted cocoa bean nibs	-	41.0	32.8	24.6	16.4	8.2
Fine whole oats	14.9	6.0	6.0	6.0	6.0	6.0
Rice flakes	24.6	10.0	10.0	10.0	10.0	10.0
Rolled oats	24.6	10.0	10.0	10.0	10.0	10.0
Chia seed	4.9	2.0	2.0	2.0	2.0	2.0
Honey	9.0	9.0	9.0	9.0	9.0	9.0
Cane molasses	11.0	11.0	11.0	11.0	11.0	11.0
Brown sugar	6.0	6.0	6.0	6.0	6.0	6.0
Soy lecithin	1.8	1.8	1.8	1.8	1.8	1.8
Salt	0.2	0.2	0.2	0.2	0.2	0.2
Water	3.0	3.0	3.0	3.0	3.0	3.0

**Table 2:** Characterization of cocoa beans from different origins.

	Origin	
	Bahia	Espírito Santo
Fermentation Index	1.31± 0.026 ^b^	1.48± 0.031 ^a^
pH	5.03 ± 0.021 ^a^	4.85± 0.014 ^b^
Moisture (%)	6.11± 0.090 ^b^	6.63 ± 0.212 ^a^

Values are media ± standard deviation. Different letters indicate significant differences at *p* < 0.05.

**Table 3:** Phenolic and total flavonoid content in cocoa beans.

	**Cocoa Treatment**	**Origin**	
		**Bahia**	**Espírito Santo**
Total phenolic (mg GAE/100 g of cocoa beans)	Unroasted	2489.54 ± 227.18 ^Aa^	1523.16 ± 56.22 ^Ab^
	Roasted	783.79 ± 64.77 ^Bb^	1161.02 ± 96.20 ^Ba^
Total flavonoid (mg de QE/100 g of cocoa beans)	Unroasted	46.78 ± 4.19 ^Aa^	24.36 ± 3.03 ^Ab^
	Roasted	21.96 ± 1.69 ^Ba^	22.58 ± 5.06 ^Aa^

Averages followed by the same uppercase letters in the column and lowercase letters in the row do not differ from each other according to Tukey’s test (*p* > 0.05).

**Table 4:** Antioxidant activity of cocoa beans.

	Cocoa Treatment	Origin	
		Bahia	Espírito Santo
Antioxidant activity ABTS (µM TEAC/g)	Unroasted	1376.99 ± 177.68 ^Aa^	959.79 ± 39.50 ^Ab^
	Roasted	434.91 ± 6.84 ^Bb^	658.24 ± 32.90 ^Ba^
Antioxidant activity DPPH (µM TEAC/g)	Unroasted	1266.91 ± 202.62 ^Aa^	708.99 ± 30.01 ^Ab^
	Roasted	413.80 ± 6.52 ^Ba^	601.49 ± 18.84 ^Aa^
Antioxidant activity FRAP (µM FSE/g)	Unroasted	417.05 ± 33.23 ^Aa^	207.97 ± 4.12 ^Ab^
	Roasted	290.31 ± 15.84 ^Ba^	161.26 ± 2.54 ^Bb^

Averages followed by the same uppercase letters in the column and lowercase letters in the row do not differ from each other according to Tukey’s test (*p* > 0.05).

**Table 5:** Concentration of the compounds identified in cocoa beans (expressed in ng/g of cocoa).

Compound	Cocoa Treatment	Origin		
		Bahia	Espírito Santo	Mean
Epicatechin	Unroasted	13,466.28 ± 2002.70	14,484.70 ± 598.16	13,975.49 ± 1342.36 ^A^
	Roasted	11,090.03 ± 286.65	12,409.42 ± 942.93	11,749.72 ± 950.80 ^B^
	Mean	12,278.15 ± 1801.81 ^a^	13,447.06 ± 1360.60 ^a^	
Catechin	Unroasted	1551.12 ± 181.18	1718.57 ± 210.66	1634.85 ± 187.30 ^A^
	Roasted	1547.47 ± 119.45	1853.56 ± 226.57	1700.52 ± 230.43 ^A^
	Mean	1549.30 ± 125.31 ^a^	1786.07 ± 194.88 ^a^	
Quercetin	Unroasted	929.72 ± 11.28	2090.22 ± 156.40	907.51 ± 105.93 ^B^
	Roasted	885.31 ± 177.66	2129.28 ± 88.74	2109.75 ± 106.24 ^A^
	Mean	1509.97 ± 676.10 ^a^	1507.30 ± 727.30 ^a^	
Caffeine	Unroasted	549.41 ± 62.03	548.64 ± 7.83	549.03 ± 36.10 ^A^
	Roasted	396.65 ± 36.86	499.12 ± 78.85	447.88 ± 77.62 ^B^
	Mean	473.03 ± 97.54 ^a^	523.88 ± 53.95 ^a^	
Theobromine	Unroasted	613.03 ± 146.41	732.20 ± 125.93	672.61 ± 131.02 ^A^
	Roasted	548.98 ± 48.91	610.44 ± 41.24	579.71 ± 51.22 ^A^
	Mean	581.00 ± 96.49 ^a^	671.32 ± 103.90 ^a^	
Caffeic acid	Unroasted	9.75 ± 2.09	8.79 ± 0.68	9.27 ± 1.39 ^A^
	Roasted	6.02 ± 0.70	6.65 ± 1.20	6.34 ± 0.88 ^B^
	Mean	7.89 ± 2.50 ^a^	7.72 ± 1.47 ^a^	
p-Coumaric acid	Unroasted	1.56 ± 0.13	2.39 ± 0.17	1.98 ± 0.50 ^A^
	Roasted	1.85 ± 0.08	2.80 ± 0.37	2.32 ± 0.60 ^A^
	Mean	1.70 ± 0.19 ^b^	2.60 ± 0.33 ^a^	

Results were expressed as means ± standard deviation. Means followed by the same lowercase letters in the rows and uppercase letters in the columns do not differ at 5% probability by the Tukey test.

**Table 6:** Colorimetric analysis of cereal bars with cocoa nibs added.

Origin of Cocoa/Formulations		Colorimetric Parameters					
		L	a*	b*	h*	C*	∆E*
Control		59.80 ± 0.010 ^a^	1.87 ± 0.015 ^f^	11.81 ± 0.012 ^a^	1.41 ± 0.001 ^a^	11.96 ± 0.009 ^a^	-
Bahia	F0	30.23 ± 0.036 ^f^	3.59 ± 0.032 ^a^	8.01 ± 0.031 ^e^	1.15 ± 0.005 ^d^	8.77 ± 0.015 ^d^	-
	F20	34.00 ± 0.006 ^d^	3.30 ± 0.017 ^b^	9.90 ± 0.023 ^d^	1.25 ± 0.002 ^c^	10.44 ± 0.020 ^c^	4.23 ± 0.061 ^c^
	F40	32.01 ± 0.012 ^e^	2,88 ± 0.015 ^e^	6.51 ± 0.021 ^f^	1.15 ± 0.002 ^d^	7.11 ± 0.022 ^e^	2.43 ± 0.008 ^d^
	F60	38.17 ± 0.020 ^b^	3.05 ± 0.006 ^d^	10.01 ± 0.012 ^c^	1.28 ± 0.001 ^b^	10.46 ± 0.009 ^c^	8.21 ± 0.022 ^a^
	F80	36.84 ± 0.015 ^c^	3.32 ± 0.017 ^c^	10.49 ± 0.070 ^b^	1.27 ± 0.003 ^b^	10.98 ± 0.062 ^b^	7.07 ± 0.046 ^b^
Control		59.80 ± 0.010 ^A^	1.87 ± 0.015 ^D^	11.81 ± 0.012 ^B^	1.41 ± 0.001 ^A^	11.96 ± 0.009 ^B^	-
Espírito Santo	F0	35.50 ± 0.025 ^F^	3.83 ± 0.017 ^B^	7.28 ± 0.038 ^F^	1.09 ± 0.010 ^E^	8.22 ± 0.029 ^E^	-
	F20	37.73 ± 0.497 ^E^	3.89 ± 0.121 ^B^	9.44 ± 0.149 ^E^	1.18 ±0.016 ^D^	10.21 ± 0.096 ^D^	3.12 ± 0.490 ^D^
	F40	39.01 ± 0.012 ^D^	3.63 ± 0.017 ^C^	10.00 ± 0.023 ^D^	1.22 ± 0.002 ^C^	10.64 ± 0.016 ^C^	4.45 ± 0.034 ^C^
	F60	40.83 ± 0.015 ^B^	4.22 ± 0.006 ^A^	12.56 ± 0.020 ^A^	1.25 ± 0.001 ^B^	13.25 ± 0.019 ^A^	7.51 ± 0.021 ^A^
	F80	40.12 ± 0.006 ^C^	3.77 ± 0.021 ^BC^	11.37 ± 0.025 ^C^	1.25 ± 0.002 ^B^	11.98 ± 0.022 ^B^	6.17 ± 0.045 ^B^

Values are means ± standard deviation. Means followed by different lowercase letters (Bahia) and uppercase letters (Espírito Santo) in the same column indicate a significant differences at *p* < 0.05.

**Table 7:** Hardness and water activity (aa) in cereal bars with nibs from Bahia.

	Hardness (N)	aa
Control	6.83 ± 1.52 ^AB^	0.5670 ± 0.0006 ^A^
F0	5.51 ± 1.25 ^B^	0.5690 ± 0.0077 ^A^
F20	3.14 ± 3.47 ^B^	0.5751 ± 0.0098 ^A^
F40	5.70 ± 3.74 ^B^	0.5679 ± 0.0056 ^A^
F60	9.20 ± 1.73 ^AB^	0.5709 ± 0.0023 ^A^
F80	12.66 ± 4.12 ^A^	0.5694 ± 0.0075 ^A^

Averages followed by the same uppercase letters in the column do not differ from each other according to the Tukey test (*p* > 0.05).

## Data Availability

The original contributions presented in the study are included in the article, further inquiries can be directed to the corresponding author.
